# Declining Mortality Rate of Hospitalised Patients in the Second Wave of the COVID-19 Epidemics in Italy: Risk Factors and the Age-Specific Patterns

**DOI:** 10.3390/life11090979

**Published:** 2021-09-17

**Authors:** Antonella D’Arminio Monforte, Alessandro Tavelli, Francesca Bai, Daniele Tomasoni, Camilla Falcinella, Roberto Castoldi, Diletta Barbanotti, Giovanni Mulè, Marina Allegrini, Elisa Suardi, Daniele Tesoro, Gianmarco Tagliaferri, Debora Mondatore, Matteo Augello, Andrea Cona, Tomaso Beringheli, Nicole Gemignani, Matteo Sala, Benedetta Varisco, Francesco Molà, Sofia Pettenuzzo, Lorenzo Biasioli, Alessandro Copes, Lidia Gazzola, Ottavia Viganò, Camilla Tincati, Anna De Bona, Teresa Bini, Giulia Marchetti

**Affiliations:** Institute of Infectious Diseases, Department of Health Science, ASST Santi Paolo e Carlo, University of Milan, 20142 Milan, Italy; Alessandro.tavelli@gmail.com (A.T.); francesca.bai@unimi.it (F.B.); daniele.tomasoni@unimi.it (D.T.); camilla.falcinella@unimi.it (C.F.); roberto.castoldi@unimi.it (R.C.); diletta.barbanotti@unimi.it (D.B.); giovanni.mule@unimi.it (G.M.); marina.allegrini@unimi.it (M.A.); elisa.suardi@asst-santipaolocarlo.it (E.S.); daniele.tesoro@unimi.it (D.T.); Gianmarco.tagliaferri@unimi.it (G.T.); Debora.mondatore@unimi.it (D.M.); matteo.augello@unimi.it (M.A.); andrea.cona@unimi.it (A.C.); Tomaso.beringheli@unimi.it (T.B.); nicole.gemignani@unimi.it (N.G.); Matteo.sala1@unimi.it (M.S.); benedetta.varisco@unimi.it (B.V.); francesco.mola@unimi.it (F.M.); sofia.pettenuzzo@unimi.it (S.P.); Lorenzo.biasioli@unimi.it (L.B.); alessandro.copes@unimi.it (A.C.); lidia.gazzola@asst-santipaolocarlo.it (L.G.); ottavia.vigano@asst-santipaolocarlo.it (O.V.); camilla.tincati@unimi.it (C.T.); Anna.debona@asst-santipaolocarlo.it (A.D.B.); teresa.bini@unimi.it (T.B.); giulia.marchetti@unimi.it (G.M.)

**Keywords:** COVID-19, hospitalised patients, age, in-hospital mortality rate, first and second wave of epidemics

## Abstract

Background: Mortality rate from COVID-19 in Italy is among the world’s highest. We aimed to ascertain whether there was any reduction of in-hospital mortality in patients hospitalised for COVID-19 in the second-wave period (October 2020–January 2021) compared to the first one (February–May 2020); further, we verified whether there were clusters of hospitalised patients who particularly benefitted from reduced mortality rate. Methods: Data collected related to in-patients’ demographics, clinical, laboratory, therapies and outcome. Primary end-point was time to in-hospital death. Factors associated were evaluated by uni- and multivariable analyses. A flow diagram was created to determine the rate of in-hospital death according to individual and disease characteristics. Results: A total of 1561 patients were included. The 14-day cumulative incidence of in-hospital death by competing risk regression was of 24.8% (95% CI: 21.3–28.5) and 15.9% (95% CI: 13.7–18.2) in the first and second wave. We observed that the highest relative reduction of death from first to second wave (more than 47%) occurred mainly in the clusters of patients younger than 70 years. Conclusions: Progress in care and supporting therapies did affect population over 70 years to a lesser extent. Preventive and vaccination campaigns should focus on individuals whose risk of death from COVID-19 remains high.

## 1. Introduction

In the last 2-year period SARS CoV-2 pandemics has caused over 4.2 million deaths worldwide [1], causing major socio-economic and health disruptions worldwide. The pandemics has spread all over Europe at different waves, the first one occurring in March–May 2020, the second one in October–January 2021 and the third one in March–May 2021. Finally, a fourth wave including highly contagious virus variants is ongoing all over the world. While the first wave was unexpected and resulted in sudden dramatic changes to health system in different countries, the second one was foreseeable and hospitals were already organised to host a number of COVID-19 in ordinary, subacute and ICU beds.

Mortality rates vary widely according to different settings and in different parts of the world [2,3]. This high variability might depend on different factors, first of all the reference population, including either only hospitalised or also out-patients, but also the health setting including the availability of ICU beds, and individual variables, such as socioeconomical status [3]; all these factors might contribute to disentangle differences on COVID-19 fatality rates in different settings.

In this context, Italy was the first European country for number of deaths well above the number of 100,000 [4]. Importantly, most deaths were in Lombardy, accounting for 49% of the country’s total [5]. Given this unexplained high frequency in this region, we aimed at understanding any possible reasons behind such trend.

The aims of this study are to ascertain any differences in in-hospital mortality rates according to the two waves periods, and to verify whether the contribution of patients’ characteristics and disease presentation to the outcome has a different weight in the first and second waves.

## 2. Materials and Methods

The prospective observational cohort includes all ≥18 years old patients hospitalised for confirmed SARS CoV-2 symptomatic infection (positive RT-PCR from nasopharyngeal or broncho-alveolar swab) from 24 February 2020 to 31 January 2021 at San Paolo University hospital, in Milan. Patients who died in emergency room within 24 h and patients not hospitalised were not included.

We divided the whole period into three groups: from 24 February to 31 May 2020 (first period), from 1 June to 30 September 2020 (second period) and from 1 October 2020 to 31 January 2021 (third period).

Data were entered into an electronic database, including: demographics; risk factors for SARS CoV-2; comorbidities; age-unadjusted Charlson comorbidity index [6]; symptoms; laboratory examinations at admission. Disease severity at admission was classified as mild (no pneumonia); moderate (pneumonia by X-ray; RR > 26/min; SO_2_ > 96% in room air; PaO_2_/FiO_2_ > 300 mmHg); severe (RR < 24/min; SO_2_ < 92%; PaO_2_/FiO_2_ 100–300 mmHg); critical disease (PaO_2_/FiO_2_ < 100 mmHg).

The highest intensity of ventilation during hospitalisation was recorded as: no need; low/high flow supplemental oxygen by nasal cannula/face mask; continuous positive airway pressure device (cPAP); mechanical non-invasive (NIV); or invasive ventilation (IV).

Use of remdesivir or other antivirals, immunomodulatory agents (tocilizumab, sarilumab, ruxolitinib, baricitinib) and high-dose corticosteroids (dexamethasone and methylprednisolone) were also collected. Primary endpoint was time to in-hospital death, discharge from hospital was analysed as a competing event by competing risks analysis.

Continuous variables are presented as median and interquartile ranges (IQR), categorical variables are presented as frequency and percentage. Difference according to calendar period of admission are evaluated by Kruskal–Wallis and Chi-square tests, as appropriate.

In-hospital mortality has been evaluated by competing risks analysis, using cumulative incidence function (CIF). In our study, discharge from hospital after recovery is considered a competing risk for in-hospital mortality, whereas in standard survival analyses (i.e., Kaplan Meier curves), patients who recover are censored. This censoring violates the assumption of noninformative censoring, as the recovered and discharge patients are not representative of those who are still admitted to the hospital in terms of their risk of dying. Censoring patients induce bias and overestimate the incidence of death.

A proportional sub-distribution hazard (SHR) model by Fine and Gray has been fitted to estimate the effect of calendar period and other covariates on CIFs in-hospital death. Pepe-Mori test was used to compare equality of CIFs across subgroups (waves of epidemic). A sub-group analysis has been performed using the subpopulation of patients severe/critical at hospital admission.

Two different sets of covariates, chosen a priori, has been used in the adjusted analyses, Model 1 does not include data on therapy used for COVID-19 while Model 2 includes as covariates the anti-COVID19 regimen used (remdesivir, corticosteroids, immunomodulators). Actually, both remdesivir and desamethasone were demonstrated to be effective after the first wave, on May 2020 for remdesivir and on July 2020 for desamethasone. Thereafter, they became the regimen of choice. Of note, remdesivir should be used only within 10 days from the onset of symptoms.

We have also investigated the in-hospital mortality by age strata (<70 and ≥70 years old).

Moreover, we have classified the participants in 16 sub-groups/clusters according to age (<70 and ≥70 years), sex (male and female), COVID-19 severity at admission (mild/moderate vs. severe/critical) and burden of comorbidities (age-unadjusted CCI <2 and ≥2). We then calculated the marginal probabilities of in-hospital death by fitting a logistic regression model with age, sex, age-unadjusted CCI, disease severity, LDH, lymphocytes count, CRP, D-dimer, and obesity as covariates without interactions and estimated the probability of in-hospital death in two calendar periods (February/May 2020 and October-2020/January-2021) according to the 16 participant’s clusters. The predicted probabilities of in-hospital death by calendar period for each cluster has been plotted to evaluate the overall and relative changes.

All the statistical analyses have been performed using Stata (version 14.0, StataCorp, College Station, TX, USA)

## 3. Results

A total of 1683 patients were hospitalised for COVID-19 at San Paolo Hospital from 24 February to 31 January 2021: 556 (33.0%) between February and May (first wave), 1005 (59.7%) between October 2020 and January 2021 (second wave), and 122 (7.2%) in the interweaves period (June–September); these last patients were not included in the analyses, due to the low number and different characteristics (Supplementary Table S1). Demographic and clinical characteristics of the remaining 1561 patients are reported in Table 1; differences in patients’ and clinical characteristics were examined according to the two calendar periods (waves of epidemics). Second-wave patients were older (second vs. first wave: median age 72 years-IQR 57–81 vs. 66 years-IQR 55–78, *p* < 001), suffering from more comorbidities (median age-unadjusted Charlson index second vs. first wave: 1 point-IQR 0–2 vs. 0 points-IQR 0–2. *p* = 0.003), and displayed a less severe SARS CoV-2-related disease (no pneumonia: 11.6% vs. 5.6%; *p* < 001). A number of disease-related symptoms and signs were different according to the different calendar periods. Noteworthy, in the second calendar period patients were referred to hospital earlier, a median of 5 days (IQR: 3–8) vs. 7 days (IQR: 3.10) from the beginning of symptoms. According to new evidence from trials remdesivir, corticosteroids and biological agents were all used more frequently in the second-wave patients. In-hospital death occurred in 178 (32%) patients belonging to the first wave and 245 (24.4%) patients belonging to the second-one (*p* < 001) (see Table 1).

The 14-day cumulative incidence of in-hospital death by competing risk regression was of 24.8% (95% CI: 21.3–28.5) and 15.9% (95% CI: 13.7–18.2) in the first and second wave, respectively (pepe-mori test *p*: < 001).

Looking to possible factors associated to in-hospital death by multivariable models, both considering in the model the use of drugs or not, patients belonging to the second wave showed a significant reduction of risk as compared to the first-wave patients (model 1 not considering drugs: ASHR 0.59. 95% CI: 0.48–0.74; model 2 including drugs: ASHR 0.61–95% CI: 0.47–0.78). Other independent variables associated with higher risk of in-hospital death were age (Model1: ASHR: 1.66–95% CI: 1.52–1.82), male sex (ASHR: 1.24–95% CI: 1.00–1.54). Diseases related parameters, such as Charlson Index, pro-inflammatory markers and disease severity at admission were all associated with the risk of in-hospital death (Table 2).

Data were confirmed in the subgroup of patients with critical disease at admission; in these patients the association remdesivir+desamethasone was associated with a significant reduction of risk of in-hospital death (ASHR: 0.53–95% CI: 0.34 -0.82; *p* = 005) (Supplementary Table S2).

To address the second aim of our study, we explored whether the reduction in in-hospital death in the second-wave patients was obtained mainly in selected clusters of patients. We therefore grouped the patients according to several characteristics: sex (M vs. F), age (≥70 vs. <70), Charlson ≥2 vs. <2), COVID-19 severity at presentation (mild/moderate vs. severe/critical) and obtained 16 different groups; we then compared the differences of in-hospital deaths (second vs. first wave) accordingly.

**Figure 1:**
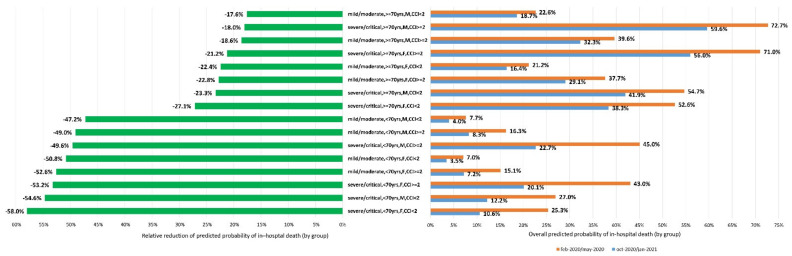
Marginal predictions of in-hospital death by sub-groups in first and second wave of epidemic (right panel) and relative reduction during the second wave (left panel). yrs = years; F = females; M = males; CCI = age unadjusted Charlson Comorbidity Index.

In all cases there was a reduction in the predictive probability of death in the second wave as compared to the first one, but it is impressive to note that patients younger than 70 benefitted with more than 45% of relative reduction, whereas patients older than 70 showed always less than 30% reduction of in-hospital death. Inside these two groups, there was a gradient of benefit according to Charlson, sex and severity of COVID-19 at presentation; being man, older than 70, with Charlson <2 and mild/moderate disease, the group with less benefit (17.6% of in-hospital death reduction) and younger than 70, females, with Charlson <2 and severe/critical disease, the group benefiting more (in-hospital death reduction by 58%). Data on reduction of predicted probability of in-hospital death in the second compared to the first wave, and of the overall predicted probability death in the individual groups according to the period are shown in Figure 1 (left and right part, respectively).

Given these findings, we analysed the probability of reduction of in-hospital death according to period separately, in patients younger and older than 70. After adjusting for all the variables listed in Table 2, patients younger than 70 showed 72% lower risk of death in the second wave (ASHR 0.28, 95% CI: 0.16–0.48), while patients older than 70 showed only 26% of risk reduction (ASHR 0.74, 95% CI: 0.56–0.99).

## 4. Discussion

In our study population including over 1500 patients hospitalised for COVID-19 we demonstrated a significant decrease of the mortality rate between the first and second wave of the epidemics, albeit with people aged over 70 years benefiting to a lesser extent from this risk reduction. More specifically, the percentage of mortality reduction varied from 17.6% in the cluster of males, older than 70, with mild/moderate COVID-19 disease and Charlson index below 2 (in-hospital mortality rate from 22.6% in the first wave to 18.7% in the second one), to 58% in the cluster of females, younger than 70, with severe/critical disease and Charlson index below 2 (in-hospital mortality rate from 25.9% in the first wave to 10.6% in the second one). It is noteworthy that a specific cluster of patients composed of men, above 70, with critical/severe disease and Charlson >2 showed a very high in-hospital mortality rate in both calendar periods, with only slight reduction between the first and the second wave (72.7% to 59.6%).

More specifically, the 1005 patients hospitalised in the second-wave period showed important differences compared to the 556 ones hospitalised in the first wave, the former being older and affected by more comorbidities and thus more likely candidates to a worse outcome. On the other hand, they showed the same severity of disease at admission and advantaged by more effective therapeutic support, such as remdesivir and corticosteroids, as the data from clinical trials became available meanwhile [7,8].

Importantly, we excluded from the analyses all the patients dying in emergency room within 24 h but also the high number of patients who showed up at our emergency rooms but ended up not being hospitalised as they were suffering from a mild disease. The exclusion of this particular group may be among the explanatory factors behind the very high rates of in-hospital deaths in the study setting. Further, the small percentage (19.5%, 48 cases) of patients hospitalised in spite of not suffering from COVID-19-related pneumonia included mostly patients from long-term care facilities, with a high load of comorbidities. Nonetheless, the number of deaths in our hospital was also higher than the other cohorts in the Milan area, showing 23% of fatality rate [9] but it should be emphasised than our population was the oldest one and with highest percentages of patients with at least two comorbidities (44% vs. 19%).

Hoffmann and Wolf [10] showed very well that mortality rate relates to age, and that Italy, UK and France are those countries with the highest mortality rates and the oldest populations. Obviously, there might have been a selection bias in reporting cases and deaths by different countries, but the authors underline that any other speculations on different mortality rates across the countries, such as genetics, viral and socio-economic factors [11,12,13] should take into account age as a main driver of worse outcome in COVID-19.

Predictive factors of worse outcome other than age and comorbidities have been already addressed in our cohort [14] and are confirmed in this updated analysis with a more than doubled population. Being older, with high comorbidity load, high inflammatory markers at hospital admission, with severe/critical COVID-19 were confirmed to be associated with a worse outcome. Further, in this larger series, females were associated to a better outcome, as already reported by other authors [15,16].

To underline, we excluded more recent periods from the analyses as possible variants, in detail B.1.617.2, the so called ‘delta variant’, known to be more contagious but probably less aggressive [17], might conditionate outcome, thus confounding our analyses.

Our study has several limitations: first, we did not collect the number of patients excluded as dying within 24 h from admission or those not requiring hospitalisation. Second, a number of variables that could be considered uncounted confounding factors are not adequately collected, most notably obesity [18]. This last variable seems to be not equally distributed among the two waves’ patients, and might have affected the estimates of the risk of death. Third, even if we have adjusted our analyses for therapy intervention, the observational nature of our study cannot allow to completely adjust for as in a trial setting.

## 5. Conclusions

In conclusion, we observed a sharp reduction of in-hospital deaths according to the first two waves of SARS CoV-2 epidemics, but males aged above 70 showed less benefit of improved survival overtime and thus should be the focus of preventive campaigns and of vaccination programs.

## 6. Patents

All patients hospitalised for COVID-19 give their consent to treatment of data related to their illness by anonymous way.

## Figures and Tables

**Table 1:** Demographic, clinical and laboratory characteristics and outcomes of 1561 patients according to the period of SARS CoV-2 epidemics.

	February 2020–June 2020		October 2020–January 2021		Total		*p*
	*N* = 556 (35.6)		*N =* 1005 (64.4)		*N =* 1561 (100.0)		
**Age, years, median (IQR)**	66	55–8	72	57–81	70	56–81	0.001
Age, >70 years, n (%)	249	44.8	547	54.0	796	51.0	<0.001
**Sex, Male**	356	64.0	635	63.2	991	63.5	0.740
**Italian**	442	79.5	808	81.2	1250	80.6	0.414
**Ethnicity**							0.044
Caucasian	460	82.7	836	83.9	1296	83.5	
Latin/Hispanic	45	8.1	60	6	105	6.8	
Black	8	1.44	4	0.4	12	0.8	
Asian	16	2.9	45	4.5	61	3.9	
Other	27	4.9	52	5.2	79	5.1	
**Epidemiology, n (%)**							<0.001
Close contact	84	15.1	50	5	134	8.6	
Healthcare workers	43	7.7	10	1	53	3.4	
Hospitalisation	31	5.6	171	17	202	12.9	
Long-term care facility	64	11.5	48	4.8	112	7.2	
Other/Unknown	334	60.1	726	72.2	1060	67.9	
**Smoking, n (%)**							<0.001
Never smoker	54	9.7	33	3.3	87	5.6	
Former smoker	57	10.3	72	7.1	57	72	
Actual smoker	13	2.3	47	4.7	60	3.8	
Unknown	432	77.7	853	84.9	1285	82.3	
**Obesity, n (%)**							<0.001
No	206	37.1	139	13.8	345	22.1	
Yes	89	16	107	10.7	196	12.6	
Unknown	261	46.9	759	75.5	1020	65.3	
**BMI, kg/m^2^, median (IQR)**	27.4	23.9–31.5	26.2	23.5–30.7	26.7	23.7–31.2	0.194
**Hypertension, n (%)**	260	46.8	514	51.1	774	49.6	0.097
**Stroke, n (%)**	49	8.8	99	9.9	148	9.5	0.503
**CPD, n (%)**	48	8.6	97	9.7	145	9.3	0.507
**IMA, n (%)**	72	13	175	17.4	247	15.8	0.021
**Diabetes, n (%)**	100	18	224	22.3	324	20.8	0.045
**Cerebrovascular diseases, n (%)**	49	8.8	99	9.9	148	9.5	0.503
**Cardiovascular diseases n (%)**	154	27.7	341	33.9	495	31.7	0.011
**COPD/Asthma, n (%)**	79	14.2	137	13.6	216	13.8	0.752
**Cancer (last 5 years), n (%)**	39	7	88	8.8	127	8.1	0.228
**CKD, n (%)**	44	7.9	84	8.4	128	8.2	0.759
**Rheumatological Diseases, n (%)**	15	2.7	12	1.2	27	1.7	0.029
**Peripheric vascular diseases, n (%)**	53	9.5	63	6.3	116	7.4	0.019
**HIV, n (%)**	4	0.7	11	1.1	15	1	0.467
**Chronic liver disease, n (%)**	20	3.6	44	4.9	64	4.1	0.456
**Age Unadjusted Charlson score, median (IQR)**	0	0–2	1	0–2	1	0–2	0.003
**Age Adjusted Charlson score, median (IQR)**	3	1–5	4	2–5	3	1–5	<0.001
**Signs and symptoms at admission**							
Fever	475	85.4	698	69.5	1173	75.1	<0.001
Dyspnoea	308	55.4	492	49	800	51.3	0.015
Cough	276	50	332	33	608	39	<0.001
Fatigue	89	16	150	14.9	239	15.3	0.570
GI Symptoms	80	14.4	115	11.4	195	12.5	<0.001
Erythromelalgia	29	5.2	45	4.5	74	4.7	0.511
Chest pain	26	4.7	36	3.6	62	4	0.289
Anosmia/dysgeusia	18	3.2	57	5.67	75	4.8	0.031
Syncope/Pre-syncope	10	1.8	48	4.8	58	3.72	0.003
**COVID Severity at admission, n (%)**							<0.001
No pneumonia	31	5.6	117	11.6	148	9.5	
Mild	254	45.7	394	39.2	648	41.5	
Severe	253	45.5	468	46.6	721	46.2	
Critical	18	3.2	26	2.6	44	2.8	
**Respiratory rate at admission, breaths/min, median (IQR)**	24	20–29	20	18–24	22	18–26	<0.001
**X-ray or CT scan signs of pneumonia**	514	92.4	804	80	1318	84.4	<0.001
**Highest grade of O_2_ therapy/ventilation during hospitalisation**							<0.001
Invasive mechanical ventilation (IMV)	69	12.4	32	3.2	101	6.5	
Non-invasive mechanical Ventilation (NIV)	52	9.4	63	6.3	115	7.4	
Continuous positive airway pressure (CPAP)	157	28.2	273	27.2	430	27.6	
O_2_ low/high flows	207	37.2	479	47.7	686	44	
No O_2_ therapy	71	12.8	158	15.7	229	14.7	
**ICU admission, n (%)**	72	13	38	3.8	110	7.1	<0.001
**Laboratory parameters**							
Haemoglobin/dL, median (IQR)	13.5	12.1–14.8	13.3	11.7–14.5	13.3	11.8–14.6	0.027
CRP, mg/L, median (IQR)	60	26.8–102.2	53.4	22.3–92.9	54.9	24–95.6	<0.001
LDH, U/L, median (IQR)	296	229–393	290	231–385	292	230–389	0.614
Leukocytes count, 10^3^/uL, median (IQR)	6.58	4.93–9.18	7.31	5.26–10.11	7	5.13–9.69	0.002
Lymphocyte count, 10^3^/uL, median (IQR)	1.02	0.68–1.37	0.98	0.69–1.4	0.99	0.68–1.38	0.719
Neutrophil count, 10^3^/uL, median (IQR)	4.76	3.35–7.44	5.34	3.64–8.12	3.15	3.53–7.85	0.002
Monocyte count, 10^3^/uL, median (IQR)	0.46	0.31–0.65	0.5	0.34–0.73	0.49	0.33–0.71	0.004
Platlets,10^3^/uL, median (IQR)	204	158–263	208	161–266	206	160–265	0.443
Creatine phosphokinase, U/L, median (IQR)	94	53–185	82	51–159	86	52–166	0.052
D-Dimer,ng/mL, median (IQR)	413	247–865	350	218–660	364	226–724	0.002
ALT, U/L, median (IQR)	29	20–49	26	18–44	27	19–45	0.009
AST, U/L, median (IQR)	41	30.5–60	39	29–56	40	30–57	0.074
Creatinine, mg/dL, median (IQR)	0.9	0.7–1.2	0.9	0.7–1.2	0.9	0.7–1.2	
Procalcitonin, ng/mL, median (IQR)	0.18	0.07–0.85	0.15	0.05–0.45	0.16	0.05–0.56	0.031
Ferritin, ng/mL, median (IQR)	447	219–858	392	164–905	419	180–872	0.119
**Days from symptoms onset and hospitalisation, median (IQR)**	7	3–10	5	2–7	5	3–8	<0.001
**Pharmacological treatments**							
Azithromycin, n (%)	168	30.2	25	2.5	193	12.4	<0.001
Lopinavir/r or Darunavir/c, n (%)	133	23.9	0	0	133	8.5	<0.001
Hydroxychloroquine, n (%)	434	78.1	1	0.1	435	27.9	<0.001
Remdesivir, n (%)	9	1.6	206	20.5	215	13.8	<0.001
Heparin prophylaxis, n (%)	360	64.8	784	78	1144	73.3	<0.001
Corticosteroid treatment, n (%)	127	22.8	719	71.5	846	54.2	<0.001
Biological (Tocilizumab, Sarilumab), n (%)	44	7.9	29	2.9	73	4.7	<0.001
**Days of Hospitalisation, median (IQR)**	10	6–21	10	6–19	10	6–20	0.304
**In-hospital death, n (%)**	178	32	245	24.4	423	27.1	<0.001

**Table 2:** Factors associated with in-hospital mortality in 1561 patients hospitalised for COVID-19.

	Unadjusted				Model1 (Without Drugs)				Model2 (With Drugs)			
	SHR	95% CI		*p*	aSHR	95% CI		*p*	aSHR	95% CI		*p*
**Age, per 10 years older**	1.73	1.61	1.85	<0.001	1.66	1.52	1.82	<0.001	1.67	1.52	1.83	<0.001
**Sex, male (vs. female)**	1.08	0.89	1.32	0.438	1.24	1.00	1.54	0.045	1.26	1.01	1.56	0.040
**Obesity (BMI > 30 kg/m^2^)**												
No	1.00				1.00				1.00			
Yes	1.39	0.98	1.96	0.064	1.50	1.04	2.15	0.029	1.47	1.02	2.11	0.038
Unknown	1.44	1.12	1.86	0.005	1.33	1.01	1.74	0.040	1.30	0.99	1.70	0.060
**Charlson age unadjusted index**												
0	1.00				1.00				1.00			
1	2.61	2.00	3.42	<0.001	1.83	1.39	2.42	<0.001	1.85	1.40	2.44	<0.001
2	2.79	2.05	3.80	<0.001	1.79	1.28	2.48	0.001	1.79	1.29	2.49	0.001
≥3	4.50	3.48	5.81	<0.001	2.35	1.76	3.12	<0.001	2.30	1.72	3.07	<0.001
**LDH >300 U/L (vs *≤* 300)**	1.99	1.62	2.45	<0.001	1.52	1.21	1.91	<0.001	1.51	1.20	1.90	<0.001
**Lymphocyte < 1.00 10^3^/uL (vs ≥ 1.000)**	1.95	1.60	2.39	<0.001	1.27	1.03	1.66	0.027	1.29	1.03	1.61	0.024
**CRP > 60 mg/L (vs *≤* 60)**	2.80	2.29	3.43	<0.001	1.80	1.44	2.25	<0.001	1.80	1.43	2.25	<0.001
**D-dimer > 1.000 ng/mL (vs.** ***≤*** **1.000)**	2.27	1.80	2.85	<0.001	1.28	1.00	1.65	0.049	1.28	1.00	1.64	0.053
**Severity**												
mild/moderate	1.00				1.00				1.00			
severe	2.75	2.23	3.39	<0.001	1.96	1.56	2.46	<0.001	1.98	1.57	2.50	<0.001
critical	9.09	6.07	13.60	<0.001	5.19	3.43	7.86	<0.001	5.18	3.45	7.77	<0.001
**anti-COVID-19 regimen**												
None	1.00								1.00			
Immunomodulators only	1.0481	0.53	2.07	0.892					0.66	0.30	1.49	0.320
Immunomodulators + Corticosteroids + Remdesivir	0.5717	0.16	2.04	0.388					0.88	0.24	3.18	0.841
Immunomodulators + Corticosteroids	1.3293	0.75	2.35	0.327					1.24	0.66	2.32	0.505
Remdesivir	0.4228	0.14	1.28	0.127					0.47	0.15	1.48	0.197
Corticosteroids	1.0591	0.86	1.30	0.586					0.98	0.76	1.28	0.902
Corticosteroids + Remdesivir	0.6977	0.50	0.98	0.036					0.78	0.53	1.14	0.195
**Waves**												
March 2020–May 2020	1.00				1.00				1.00			
October 2020–January 2021	0.71	0.58	0.86	<0.001	0.59	0.48	0.74	<0.001	0.61	0.47	0.78	<0.001

## Data Availability

Upon request, the dataset can be available at our Institution.

## Supplementary Materials

The following are available online at https://www.mdpi.com/article/10.3390/life11090979/s1. Supplemental Table S1. Demographic, clinical and laboratory characteristics and outcomes of 1683 patients according to the period of SARS cov-2 epidemics. Supplemental Table S2. Factors associated with in-hospital death in severe/critical subjects at admission by fitting a fine-Gray model.
