# Supplementary material for: Declining Mortality Rate of Hospitalised Patients in the Second Wave of the COVID-19 Epidemics in Italy: Risk Factors and the Age-Specific Patterns
Source: Life (Basel). 2021 Sep 17;11(9):979. doi: 10.3390/life11090979 (PMC8464683; doi:10.3390/life11090979)
Supplement: Supplementary file 1 [file life-11-00979-s001.zip › life-1351563-supplementary.pdf]

Supplemental Table S1. Demographic, clinical and laboratory characteristics and outcomes of 1,683 patients according to the period of SARS CoV-2 epidemics

	Feb2020-May2020 N=556 (33.04)		Jun2020-Sep2020 N=122 (7.25)		Oct2020-Jan2021 N=1005 (59.71)		Total N=1683 (100.0)		p
**Age, years, median (IQR)**	66	55-78	72	54-84	72	57-81	70	56-81	<0.001
**Sex, Male, n(%)**	356	64.0	51	41.8	635	63.18	1042	61.91	<0.001
**Italian, n(%)**	442	79.5	88	72.1	808	81.21	1338	79.5	0.058
**Ethnicity, n(%)**									0.188
Caucasian	460	82.7	97	79.5	836	83.9	1393	83.16	
Latin/hispanic	45	8.1	10	8.2	60	6	115	6.9	
Black	8	1.44	1	0.8	4	0.4	13	0.78	
Asian	16	2.9	7	5.7	45	4.5	68	4.1	
Other	27	4.9	7	5.7	52	5.2	86	5.1	
**Epidemiology, n(%)**									<0.001
Close contact	84	15.1	5	4.1	50	5	139	8.3	
Healthcare workers	43	7.7	2	1.6	10	1	55	3.3	
Hospitalization	31	5.6	15	12.3	171	17	217	12.9	
RSA	64	11.5	28	23	48	4.8	140	8.3	
Other/Unknown	334	60.1	72	59	726	72.2	1132	67.3	
**Smoking, n(%)**									<0.001
Never smoker	54	9.7	4	3.3	33	3.3	91	5.4	

Former smoker	57	10.3	8	6.6	72	7.1	137	8.1	
Actual smoker	13	2.3	3	2.5	47	4.7	63	3.7	
Uknown	432	77.7	107	87.7	853	84.9	1392	82.7	
**Obesity, n(%)**									<0.001
No	206	37.1	34	27.9	139	13.8	379	22.5	
Yes	89	16	16	13.1	107	10.7	212	12.6	
Unknown	261	46.9	72	59	759	75.5	1092	64.9	
**Weight, median (IQR)**	80	68-92	75	61-80	77	67-85	78	68-89	
**Height, median (IQR)**	170	161-175	168	1600-175	170	160-175	170	160-175	0.560
**BMI, median (IQR)**	27.4	23.9-31.5	26.1	23.1-28.2	26.2	23.5-30.7	26.6	23.7-31.1	0.237
**Hypertension, n(%)**	260	46.8	62	50.8	514	51.1	836	49.7	0.244
**Stroke, n(%)**	49	8.8	13	10.7	99	9.9	161	9.6	0.731
**CPD, n(%)**	48	8.6	14	11.5	97	9.7	159	9.5	0.587
**IMA, n(%)**	72	13	19	15.6	175	17.4	266	15.8	0.068
**Diabetes, n(%)**	100	18	22	18	224	22.3	346	20.6	0.102
**Cerebrovascular Diseases, n(%)**	49	8.8	13	10.7	99	9.9	161	9.6	0.731
**Cardiovascular diseases n(%)**	154	27.7	44	36.1	341	33.9	539	32	0.025
**COPD/Asthma, n(%)**	79	14.2	15	12.3	137	13.6	231	13.7	0.849
**Cancer (last 5 years), n(%)**	39	7	13	10.7	88	8.8	140	8.3	0.306
**CKD, n(%)**	44	7.9	8	6.6	84	8.4	136	8.1	0.452
**Reumathological Diseases, n(%)**	15	2.7	2	1.6	12	1.2	29	1.7	0.091

**Periferic vascular diseases,n(%)**	53	9.5	12	9.8	63	6.3	128	7.6	0.766
**HIV, n(%)**	4	0.7	0	0	11	1.1	15	0.9	0.416
**Chronic Liver Disease, n(%)**	20	3.6	5	4.1	44	4.9	69	4.1	0.758
**Age Unadjusted Charlson score, median (IQR)**	0	0-2	1	0-2	1	0-2	1	0-2	0.002
**Age Adjusted Charlson score, median (IQR)**	3	1-5	4	1-6	4	2-5	3	1-5	0.002
**COVID Severity at admission, n(%)**									<0.001
No pneumonia	31	5.6	37	30.3	117	11.6	185	11	
Mild	254	45.7	58	47.5	394	39.2	706	42	
Severe	253	45.5	27	22.1	468	46.6	748	44.4	
Critical	18	3.2	0	0	26	2.6	44	2.6	
**PO2/FiO2 at admission, median (IQR)**	301	235-352	353	309-407	300	229-348	301	233-352	<0.001
**Respiratory rate at admission, breaths/min, median (IQR)**	24	20-29	19	16-24	20	18-24	22	18-26	<0.001
**X-ray or CT scan signs of pneumonia, n(%)**	514	92.4	76	62.3	804	80	1394	82.8	<0.001
**Signs and symptoms at admission, n(%)**									
Fever	475	85.4	54	44.2	698	69.5	1227	72.9	<0.001
Dyspnea	308	55.4	36	29.5	492	49	836	50	<0.001

Cough	276	50	27	22.1	332	33	635	37.7	<0.001
Fatigue	89	16	13	10.7	150	14.9	252	15	0.324
GI Symptoms	80	14.4	21	17.2	115	11.4	216	12.8	0.081
Arthromyalgia	29	5.2	8	6.6	45	4.5	82	4.9	0.542
Chestpain	26	4.7	1	0.8	36	3.6	63	3.7	0.116
Anosmia/dysgeusia	18	3.2	8	6.6	57	5.67	83	4.9	0.072
Syncope/Pre-syncope	10	1.8	2	1.6	48	4.8	60	3.6	0.005
**Laboratory parameters admission**									
Hemoglobin,g/dL, median (IQR)	13.5	12.1-14.8	12.8	11.2-14.6	13.3	11.7-14.5	13.3	11.8-14.6	0.028
CRP, mg/L, median (IQR)	60	26.8-102.2	25.2	6.2-67.4	53.4	22.3-92.9	53.4	22.1-94.8	<0.001
LDH , U/L, median (IQR)	296	229-393	219.5	182-282	290	231-385	287	222-380	<0.001
Leukocytes count, 10^3/uL, median (IQR)	6.58	4.93-9.18	7.37	5.52-10.1	7.31	5.26-10.11	7.04	5.15-9.76	0.003
Lymphocyte count, 10^3/uL, median (IQR)	1.02	0.68-1.37	1.31	0.94-1.9	0.96	0.69-1.4	1.01	0.69-1.43	<0.001
Neutrophil count, 10^3/uL, median (IQR)	4.76	3.35-7.44	4.93	3.44-8.02	5.34	3.64-8.12	5.12	3.52-7.86	0.008
Monocyte count, 10^3/uL, median (IQR)	0.46	0.31-0.65	0.57	0.47-0.73	0.5	0.34-0.73	0.5	0.33-0.71	<0.001
Platlets,10^3/uL, median (IQR)	204	158-263	225	172-288	208	161-266	208	161-267	0.139
Creatine phosphokinase, U/L, median (IQR)	94	53-185	70	43-155	82	51-159	85	51-166	0.020
D-Dimer, ng/mL ,median (IQR)	413	247-865	300	180-690	350	218-660	362	218-660	0.003

ALT, U/L, median (IQR)	29	20-49	21	14-34	26	18-44	27	18-44	<0.001
AST, U/L, median (IQR)	41	30.5-60	31	24.5-43	39	29-56	39	29-57	<0.001
Creatinin, mg/dL ,median (IQR)	0.9	0.7-1.2	0.8	0.7-1.1	0.9	0.7-1.2	0.9	0.7-1.2	0.867
Procalcitonin, ng/mL, median (IQR)	0.18	0.07-0.85	0.19	0.08-0.65	0.15	0.05-0.45	0.16	0.06-0.56	0.069
Ferritin, ng/mL, median (IQR)	447	219-858	281.5	124-590	392	164-905	406	174-850	0.004
**Days from symptoms onset and hospitalization, median (IQR)**	7	3-10	3	1-6	5	2-7	5	2-8	<0.001
**Days of Hospitalization, median (IQR)**	10	6-21	10	6-19	10	6-19	10	6-20	0.576
**Highest grade of O2 therapy/ ventilation, n(%)**									<0.001
Invasive Mechanical Ventilation (IMV)	69	12.4	1	0.8	32	3.2	102	6.1	
Non Invasive mechanical Ventilation (NIV)	52	9.4	3	2.5	63	6.3	118	7	
Continuous Positive Airway Pressure (CPAP)	157	28.2	25	20.5	273	27.2	455	27	
O2 low/high flows	207	37.2	42	34.4	479	47.7	728	43.2	
No O2 therapy	71	12.8	51	41.8	158	15.7	280	16.6	
**Pharmacological treatments**									
Azithromycin, n(%)	168	30.2	4	3.3	25	2.5	197	11.7	<0.001
Lopinavir/r or Darunvir/c, n(%)	133	23.9	4	3.3	0	0	137	8.1	<0.001
Hydroxychloroquine, n(%)	434	78.1	3	2.5	1	0.1	438	26.0	<0.001

Remdesivir, n(%)	9	1.6	14	11.5	206	20.5	229	13.6	<0.001
Heparin profilaxis, n(%)	360	64.8	69	56.6	784	78	1213	72.1	<0.001
Corticosteroid treatment, n(%)	127	22.8	29	23.8	719	71.5	875	51.2	<0.001
Biological (Tocilizumab, Sarilumab)	44	7.9	2	1.6	29	2.9	75	4.4	<0.001
**ICU admission, n(%)**	72	13	4	3.2	38	3.8	114	6.8	<0.001
**In-hospital death, n(%)**	178	32	16	13.1	245	24.4	439	26.1	<0.001

Supplemental Table S2. Factors associated with in-hospital death in 765 severe/critical subjects at admission by fitting a Fine-Gray model

	Unadjusted				Model1 (No drugs)				Model2 (with drugs)			
	SHR	95% CI		p	SHR	95% CI		p	SHR	95% CI		p
**Age, per 10 years older**	1.63	1.49	1.78	<0.001	1.60	1.43	1.80	<0.001	1.59	1.42	1.78	<0.001
**Sex, male (vs. female)**	0.99	0.78	1.26	0.949	1.21	0.94	1.56	0.136	1.22	0.94	1.58	0.131
**Obesiy (BMI>30 kg/m2)**												
No	1.00				1.00				1.00			
Yes	1.52	1.00	2.31	0.052	1.77	1.14	2.76	0.012	1.69	1.07	2.65	0.023
Unknown	1.62	1.17	2.23	0.003	1.52	1.09	2.12	0.014	1.46	1.03	2.07	0.036
**Charlson age unadjsuted index**												
0	1.00				1.00				1.00			
1	2.20	1.62	2.99	<0.001	1.67	1.21	2.30	0.002	1.76	1.26	2.45	0.001
2	2.03	1.41	2.92	<0.001	1.50	1.03	2.19	0.036	1.53	1.05	2.24	0.027
>=3	3.57	2.64	4.83	<0.001	2.27	1.64	3.15	<0.001	2.29	1.64	3.19	<0.001
**LDH >300 U/L**	1.41	1.10	1.82	0.007	1.48	1.13	1.92	0.004	1.47	1.13	1.93	0.004

**Lymphocye < 1.00 10^3/uL**	1.63	1.29	2.08	<0.001	1.36	1.06	1.74	0.017	1.40	1.08	1.81	0.010
**CRP >60 mg/L (vs <=60 mg/L)**	1.73	1.35	2.22	<0.001	1.66	1.27	2.16	<0.001	1.63	1.24	2.14	<0.001
**D-dimer > 1.000 ng/mL (vs. <=1.000 ng/mL)**	1.96	1.50	2.57	<0.001	1.29	0.96	1.73	0.096	1.32	0.98	1.78	0.063
**COVID-19 regimen**												
None	1.00								1.00			
Immunomodulators only	0.57	0.27	1.17	0.125					0.72	0.32	1.62	0.422
Immunomodulators+Corticosteroids+Remdesivir	0.49	0.10	2.54	0.396					0.63	0.11	3.60	0.600
Immunomodulators+Corticosteroids	0.52	0.24	1.14	0.105					0.61	0.26	1.41	0.248
Remdesivir	0.19	0.03	1.28	0.088					0.16	0.02	1.21	0.077
Corticosteroids	0.65	0.51	0.83	0.001					0.75	0.55	1.03	0.080
Corticosteroids+Remdesivir	0.44	0.29	0.65	<0.001					0.53	0.34	0.82	0.005
**Waves**												
Mar-May2020	1.00				1.00				1.00			
Oct2020-Jan2021	0.69	0.55	0.86	0.001	0.65	0.50	0.85	0.001	0.80	0.59	1.09	0.154
